# Supplementary material for: Humans treat unreliable filled-in percepts as more real than veridical ones
Source: eLife. 2017 May 16;6:e21761. doi: 10.7554/eLife.21761 (PMC5433845; doi:10.7554/eLife.21761)
Supplement: Supplementary file 1. — Empty cells indicate that the condition was not measured in this study. DOI: http://dx.doi.org/10.7554/eLife.21761.013 [file elife-21761-supp1.docx]

| **Parameter** | **Experiment 1 (95% CDI)** | **Experiment 2 (95% CDI)** | **Experiment 3 (95% CDI)** | **Experiment 4**  **(95% CDI)** | **Experiment 5**  **(95% CDI)** | **All Experiments**  **(95% CDI)** |
| --- | --- | --- | --- | --- | --- | --- |
| Location BS | 15.01% [8.49%,21.08%] | 12.50% [7.35%,17.49%] | 13.18% [6.47%,19.64%] | -7.74%  [-13.68%,-1.56%] | 14.53% [7.56%,21.09%] | 13.82%  [10.84%,16.78%] |
| Location above |  | 6.63% [0.77%,12.30%] |  |  | 5.84%  [-1.33%,13.01%] | 6.21%  [2.71%, 9.66%] |
| Location outward |  |  | 4.80% [0.58%,8.89%] |  |  | 4.91%  [1.02%,8.84%] |
| Location inward |  |  | 2.85%  [-1.10%,6.65%] |  |  | 3.01%  [-1.06%, 6.97%] |
| BS - above |  | 6.11% [1.16%,10.78%] |  |  | 8.95% [3.91%,13.85%] | 7.87%  [4.22%,11.63%] |
| BS - outward |  |  | 8.61% [0.98%,16.04%] |  |  | 9.14%  [4.63%,13.39%] |
| BS - inward |  |  | 10.51% [3.55%,17.29%] |  |  | 11.00%  [6.61%,15.40%] |
